# How to win the ovarian cancer stem cell battle: destroying the roots

**DOI:** 10.20517/cdr.2020.93

**Published:** 2020-12-22

**Authors:** Akimasa Takahashi, Linda Hong, Ilana Chefetz

**Affiliations:** ^1^The Hormel Institute, University of Minnesota, Austin, MN 55912, USA.; ^2^Department of Obstetrics and Gynecology, Shiga University of Medical Science, Otsu, Shiga 5202152, Japan.; ^3^Department of Obstetrics & Gynecology, Division of Gynecologic Oncology, Loma Linda University School of Medicine, CA 92350, USA.; ^4^Masonic Cancer Center, Minneapolis, MN 55455, USA; ^5^Stem Cell Institute, Minneapolis, MN 55455, USA; ^6^Department of Obstetrics, Gynecology and Women’s Health, Minneapolis, MN 55455, USA.

**Keywords:** Ovarian cancer, platinum resistance, cancer stem cell, chemotherapy, targeted therapy

## Abstract

Ovarian cancer has the highest mortality rate among gynecologic malignancies. The combination of cytoreductive surgery and chemotherapy is the standard regimen for the treatment of ovarian cancer. The initial treatment is usually effective, but many patients with ovarian cancer experience recurrence, and treatment options for recurrent disease remain challenging. Cancer stem cells (CSCs) are suggested to play an essential role in cancer recurrence after initial chemotherapy. Furthermore, they are of great interest as CSCs may also be involved in chemotherapy susceptibility. Thus, understanding the characteristics and mechanisms by which CSCs display resistance to therapeutic agents is important to design effective cancer treatments. In this review, we describe and discuss current therapeutic regimens for ovarian cancer, as well as the various CSC markers, association between CSCs and disease progression, correlation of CSCs with poor prognosis, enrichment of CSCs in tumor tissues following repeated chemotherapy cycles, activation of major signaling pathways following chemotherapy, and potential inhibitors that suppress these signaling cascades. In addition, clinical trials evaluating novel targeted therapies to overcome chemotherapy resistance will be reviewed. The combination of traditional chemotherapy and CSC-targeted therapy could be an effective and promising anticancer treatment for ovarian cancer. Understanding the biological properties of CSCs and the mechanism of chemotherapy resistance are critical to design and develop new therapeutic strategies to overcome CSC-associated chemotherapy resistance.

## Introduction

Ovarian cancer is the leading cause of gynecologic cancer-related death. Optimal cytoreductive surgery in combination with chemotherapy is the standard treatment for ovarian cancer. After first-line chemotherapy with carboplatin and paclitaxel, 80% of ovarian cancer patients will initially respond. However, 80% of advanced-stage patients and 20% of early-stage patients will eventually relapse. Recent tumorigenesis hypothesis suggests that cancer stem cells (CSCs) remaining in the tumor after chemotherapy are able to initiate and propagate tumors causing recurrence and chemotherapy resistance. In this review, we describe the current knowledge of ovarian cancer therapeutic options, the association between CSC expression and prognosis, chemotherapy-associated CSC enrichment, and the signaling pathways involved. Finally, we discuss the targeted cancer therapy approaches currently in clinical trials based on their effect on CSCs.

## Ovarian cancer and therapeutic options

Epithelial ovarian cancer (EOC) has the highest morbidity and mortality rate among the gynecologic malignancies in developed countries^[1]^. In the United States, an estimated 22,240 women were diagnosed with ovarian cancer in 2018, and the age-adjusted mortality rate in 2018 was 6.27 deaths per 100,000 people^[2]^. Three quarters of EOC cases are classified into four major histologic subtypes, including serous, endometrioid, clear cell, and mucinous ovarian cancer, with the remaining 25% representing rare or unspecified subtypes^[3]^. Ovarian cancer is categorized as type I and II and thought to be mediated by different signaling pathways. Type I cancers comprise low-grade serous, low-grade endometrioid, clear cell, mucinous cancers, and Brenner tumors and contain mutations in common oncogenes such as *kirsten rat sarcoma 2 viral oncogene homolog* (*KRAS*), *B-raf proto-oncogene* (*BRAF*), *erb-b2 receptor tyrosine kinase 2* (*ERBB2*), *catenin beta 1* (*CTNNB1*), *phosphatase and tensin homolog* (*PTEN*), *phosphatidylinositol 3-kinase catalytic subunit alpha isoform* (*PIK3CA*), and A*T-rich interacting domain containing protein 1A* (*ARID1A*)^[4]^. Type II cancers include aggressive malignancies, such as high grade serous carcinomas, carcinosarcoma, and undifferentiated carcinomas with mutations most notable in *tumor protein 53* (*TP53)* and breast cancer susceptibility gene (*BRCA*)^[4]^. Population-based studies have indicated that approximately 14% of ovarian cancer patients have the germline *BRCA1* and *BRCA2* mutations^[5]^. Over 75% of the ovarian cancer patients present with advanced stage disease, as defined by the spread of the disease outside the pelvis^[6,7]^. As a result, the 5-year survival rate of all ovarian cancer patients is 47%^[2]^. A combination of debulking surgery and chemotherapy is essential for the treatment of ovarian cancer. While the main purpose of primary surgery is to completely remove all macroscopically visible disease^[8]^, chemotherapy is necessary as the majority of patients will relapse despite nearly complete resection^[9]^. Platinum-based antineoplastic agents have been key therapeutic options during the past three decades^[10]^. Following first-line chemotherapy with carboplatin and paclitaxel, about 80% of ovarian cancer patients will respond and 40%-60% of patients will achieve complete remission^[11]^. Unfortunately, even for stage I or II patients, recurrence occurs in 20%-25% cases; whereas more than 80% of patients with advanced disease will recur^[12]^. The majority of patients with advanced ovarian cancer will experience disease relapse within 2 years of first-line treatment^[13]^, emphasizing an urgent unmet medical need for novel therapies. Recently, a combination of two targeted therapies for advanced ovarian cancer has been approved that includes a humanized anti-vascular endothelial growth factor (VEGF) monoclonal antibody and poly [adenosine diphosphate (ADP)-ribose] polymerase (PARP) inhibitor. Randomized clinical trials have shown significant benefits in terms of progression-free survival for concomitant use of these agents with standard chemotherapy or as single agents in maintenance regimens^[14-16]^. In particular, the presence of mutations in *BRCA1/2* genes, which play an important role in homologous recombination repair of DNA double-strand breaks, sensitize tumor cells to various PARP inhibitors (PARPi), such as olaparib, veliparib, rucaparib, niraparib, and veliparib^[17]^.

Ovarian cancer recurrence is mostly incurable; however, overall survival generally depends on platinum sensitivity. In general, patients who relapse more than 6 months after initial therapy are defined as “platinum-sensitive”, patients who relapse within 6 months are characterized as “platinum-resistant”, and patients who fail to respond or progress during initial treatment are defined as “platinum refractory”^[18]^. Patients with “platinum-sensitive” disease are treated with platinum doublet chemotherapy similarly to the initial treatment, but the vast majority of these patients will recur creating a progressive resistance to treatment, resulting in depletion of available treatment options. The main objective of treatment in recurrent disease is palliative, with a focus on controlling symptoms, prolonging survival, and improving quality of life^[19]^. Patients with recurrent ovarian cancer are commonly treated with a second line of non-platinum agents, such as paclitaxel, topotecan, gemcitabine, and pegylated liposomal doxorubicin to improve the survival prognosis of “platinum-refractory” and “platinum-resistant” patients^[20]^.

## Ovarian cancer stem cells

CSCs represent a small subset of an inherently chemoresistant population of cancer cells. In addition to the ability of self-renewal and differentiation due to ability to divide symmetrically and asymmetrically, CSCs have the ability to initiate and propagate tumors. Due to their slow cell cycle progression, CSCs are characterized by inherent resistance to standard radio- and chemotherapies^[21,22]^. Chemoresistance in recurrent ovarian cancer is associated with a higher frequency of CSCs in comparison to primary ovarian cancer^[23]^. The presence of cells with stem-like properties in cancer was first described in studies of human acute myelogenous leukemia^[24]^ in which leukemia-initiating cells were isolated based on the expression of cell surface markers CD34^+^ and CD38^+^. Subsequently, cell surface marker-based purification methods are now commonly used to isolate CSCs from heterogeneous solid tumor cells^[25,26]^.

The first CSCs in ovarian cancer were identified in the ascites of EOC patients. These cells, characterized by CD44^+^/CD117^+^ expression, developed tumors in mice continuously for several generations^[27]^. Afterwards, isolation of CSCs in ovarian cancer has been explored in various ways, including side population (SP) cells with expression of adenosine triphosphate **(**ATP)-binding cassette (ABC) transporters, the use of cell surface markers, and detection of aldehyde dehydrogenase activity (ALDH) by using the ALDEFLUOR fluorescence assisted cell sorting (FACS) method. Expression of various cell surface markers such as CD44, CD117, CD133, CD24, and epithelial cell adhesion molecule (EpCAM)^[28-37]^ has been reported in ovarian cancer. SP cells are characterized by the exclusion of the Hoechst 33342 dye through the ABC transporters, which transport a wide range of substrates such as drugs, metabolic products, nutrients, and lipids across extracellular and intracellular membranes. SP cells exhibit high self-renewal and proliferative capacity *in vitro* and possess typical CSC properties associated with tumor initiation *in vivo* and acquired resistance to chemotherapy as a result of shuffling out chemotherapeutic drugs by the ABC transporters^[38,39]^. However, isolation of SP cells has low specificity, and the purity of isolated cells is not sufficient compared to other methods due to high heterogeneity.

CD44 is a cell surface adhesion receptor and CD44^+^ cells have been reported to be present in primary and metastatic ovarian tumors as well as in cells of malignant ascites^[27,40]^. CD44^+^ cells, isolated from ascites and solid tumors, are characterized by constitutive nuclear factor-kappa B (NF-κB) activity, cytokine and chemokine production, high capacity of tumor repair and self-renewal, and resistance to conventional chemotherapy, which are unique features of CSCs^[41]^. Myeloid differentiation protein 88 (MyD88) is an adapter protein required for toll-like receptor (TLR) signal transduction as part of an inflammatory response to bacterial and viral infection. MyD88^+^ cells in ovarian cancer primary cells and cell lines are resistant to paclitaxel, a known TLR4 ligand^[42]^ and are characterized by secretion of proinflammatory cytokines. Also, a small number of CD44^+^/MyD88^+^ EOC cells can initiate tumors, suggesting CSC properties. In addition to TLR4, CD44^+^/MyD88^+^ cells express TLR2, which can be activated by injury (surgery) and chemotherapy, creating a pro-inflammatory microenvironment that further enhances ovarian CSC repair and self-renewal^[37]^. However, CD44^+^ is also expressed by many non-cancer cells in tumors, such as immune cells and vascular endothelial cells, suggesting a need to combine CD44 with additional markers to differentiate between different populations residing in tumors^[41,43]^. Thus, despite multiple studies showing that CD44^+^ cells have CSC properties, ALDH^+^/CD133^+^ double positive cells have been reported to be more abundant in recurrent tumors^[32]^. CD133^+^, which was initially identified as a CSC marker in human glioblastoma^[26]^, is one of the best characterized cell surface markers in ovarian cancer^[29,44]^. Whereas CD133^+^ ovarian CSCs have exhibited tumor initiation, self-renewal, and chemoresistance capacity, not all ovarian cancer cell lines express CD133, and a few recent studies reveal no established association between CSCs and CD133^[45,46]^. Previous reports have identified ALDH activity as the only functional marker present in all ovarian cancer cell lines^[47]^. ALDH^high^ (cells with high ALDH activity) cells detected by the ALDEFLUOR FACS assay exhibit CSC capacities, as high ALDH activity correlates with higher sphere-formation ability, tumorigenicity, and invasiveness^[48,49]^. In addition, ALDH^high^/CD133^+^ cells are capable of forming larger tumors during shorter time periods in mouse xenografts and also efficiently form three-dimensional spheres^[50]^ compared to other cell populations. Existence of ALDH^high^/CD133^+^ in debulked primary ovarian tumor specimens is associated with worse prognosis than the existence of ALDH^low^ and ALDH^high^/CD133^-^ populations^[47]^. Comparing tumor initiation capacity, we found that at least 100 CD44, CD24, CD133, ALDH, or SP single positive cells are required to initiate tumors in mouse xenograft models^[29,39,40,47,51]^; whereas fewer of CD44^+^/CD117^+^ cells, CD44^+^/CD24^+^/EpCAM^+^ cells, or CD117^+^/lineage^-^ cells^[28,31,52]^ were required to form tumors when combination of two or three markers was used to isolate cells. Importantly, only 30 ALDH^+^/CD133^+^ cells in murine models of ovarian cancer are sufficient to form tumors subcutaneously^[47]^ [Table 1]. Therefore, ALDH and CD133 are defined as a set of functionally important markers that identify ovarian CSCs.

**Table 1:** Tumorigenicity in in vivo model by ovarian cancer stem cell markers

CSC marker	Combination	Mode of CSC injection	Number of cells	Latency	Ref.
CD44		SC	1 × 10^6^	6-8 weeks	[40]
	CD117(+)	SC	1 × 10^2^	52-93 days	[28]
	CD24(+), EpCAM(+)	SC	1 × 10^2^	5 weeks	[31]
CD117	Lineage (CD2, CD3, CD10, CD16, CD31, CD64)(-)	SC	1 × 10^2^	100-128 days	[52]
CD133		SC	1 × 10^2^	99 days	[29]
	ALDH(+)	SC	2 × 10^3^	Not shown	[32]
	ALDH(+)	SC	30	Not shown	[47]
CD24		SC	5 × 10^3^	73 or 89 days	[51]
ALDH		SC	1 × 10^2^	Not shown	[47]
SP cells		IP	5 × 10^4^	30-60 days	[39]

CSC: cancer stem cell; SP: side population; ALDH: aldehyde dehydrogenase; SC: subcutaneous injection; IP: intraperitoneal injection

Nanog homeobox (NANOG), octamer-binding transcription factor 4 (OCT4), and sex-determining region Y-box 2 (SOX2) are transcription factors and stemness markers that maintain pluripotency and self-renewal of embryonic and induced pluripotent stem cells. These factors are known to be commonly expressed in ovarian CSCs^[37,53,54]^ While many CSC marker candidates have been established, we hypothesize that different sub-populations exhibit various functional roles, which suggests a need for more systematic characterization.

## Association of ovarian CSCs with poor prognosis

Various studies have identified the association between CSCs and a shorter progression-free interval and worse prognosis in ovarian cancer^[55,56]^, including a recent meta-analysis study that examined the correlation between four representative ovarian CSC marker candidates ALDH1/CD44/CD117/CD133 and prognosis^[57]^. In this meta-analysis, the increased ALDH1, CD44, and CD117 levels as assessed by immunochemistry were associated with poor prognosis, whereas CD133 was not. In addition, the overexpressed ALDH1 and CD44 were correlated with worse progression, but CD117 and CD133 were not^[57]^. Thus, CSC markers can be useful as predictive or prognostic biomarkers of ovarian cancer. In addition, high expression of EpCAM in ovarian cancer was associated with tumor recurrence and poor prognosis^[58]^. The disadvantage of this study was that only single CSC markers were examined. For instance, ALDH activity in combination with CD133 has revealed strong association with poor patient prognosis^[32,47]^. Furthermore, CD44^+^/CD24^-^ expression correlates with increased recurrence rate and reduced progression-free survival in ovarian cancer patients^[33]^. Stemness and pluripotency factors such as OCT4, nanog, and cellular myelocytomatosis oncogene (c-Myc) have been shown to be expressed in ascites and tumor samples of ovarian cancer patients^[59]^. The level of OCT4 and RNA-binding protein Lin28 have been correlated with tumor malignancy and increased tumor growth^[60]^. Additional studies revealed the role of SOX2 in tumorigenicity, migration, and invasion as well as chemoresistance^[53]^. High expression of SOX2 has been associated with a significantly reduced overall survival in ovarian cancer patients, but no association between nanog or OCT4 expression and overall survival was identified^[54]^. Similar to OCT4 and SOX2, c-Myc has characteristics of stemness and pluripotency, and is overexpressed not only in ovarian cancer but also in most types of malignant tumors^[61,62]^. In the integrated analysis of ovarian cancer by the cancer genome atlas (TCGA), c-Myc amplification was identified in 30%-60% of ovarian tumors^[63]^. This analysis revealed that disease-free survival and overall survival were decreased in ovarian cancer patients with high levels of *c-Myc* mRNA^[64]^.

## Repetitive cycles of chemotherapy and cancer stem cell enrichment

In ovarian cancer, chemotherapy decreases tumor burden but often results in enrichment of ovarian CSCs in residual tumors^[65]^. Increased mRNA levels of CD44, EpCAM, and Oct4 have been observed in ascites-derived cells following multiple doses of cytotoxic chemotherapeutic agents^[66]^. In studies that compared CSC markers in matched primary and recurrent tumors, the expression of ALDH1A1, CD44, and CD133 markers was higher in recurrent tumors compared to primary tumors^[67]^. In an experimental study, ovarian cancer cells treated with the combination of paclitaxel and carboplatin exhibited enrichment of CD117^+^ and CD133^+^ CSCs^[68]^. Furthermore, short-term administration of cisplatin and paclitaxel resulted in the enrichment of the OCT4 and CD117^+^ ovarian CSCs *in vivo* as assessed by the quantitative polymerase chain reaction (qPCR)^[69]^. In another experiment, cell cycle-arrested cells demonstrated overexpression of OCT4, nestin, CD117, and CD44 markers following cisplatin treatment^[70]^.

Recently, the Food and Drug Administration (FDA) approved several PARPi for the treatment of recurrent ovarian cancer in patients with BRCA-proficient or -deficient tumors. While analysis of CSCs in clinical samples following PARPi has to be determined, *in vitro* and *in vivo* studies exhibited enriched CD133^+^ and CD117^+^ ovarian CSCs following PARP inhibition^[71]^. These data suggest that CSC enrichment can occur both after conventional antineoplastic chemotherapy as well as novel targeted therapies, suggesting a need for novel combination regimens that include CSC-targeted therapies.

## Repetitive cycles of chemotherapy and activation of signaling pathways

Several key signaling pathways have been associated with stemness and self-renewal, including wingless-related integration site (Wnt)/β-catenin, Hedgehog, Notch, phosphatidylinositol 3-kinase (PI3-K)/phosphatase and tensin homolog deleted chromosome 10 (PTEN), and NF-κB pathways. Upregulation of c-kit, a stem cell-associated receptor tyrosine kinase, causes upregulated mRNA expression of genes from the Wnt/β-catenin pathway, subsequently leading to increased expression of ABC subfamily G member2 (ABCG2) in ovarian CSCs, suggesting drug efflux from tumors^[72]^. Also, intraperitoneal administration of taxane in an *in vivo* mouse model triggered activation of Wnt signaling in tumor specimens and enriched expression of CSCs. Wnt antagonists, vantictumab and ipafricept, strongly block paclitaxel-mediated mitosis and promote mitotic cell death, effectively alleviating CSC expression. As a result, a combination regimen comprised of paclitaxel and Wnt inhibitors effectively reduced CSC content and tumor growth^[73]^. Activation of smoothened (SMO), patched (PICH), and hedgehog glioma-associated oncogene 1 (GLI1) in the Hedgehog signaling pathway has been reported in EOC cells. In particular, SMO and GLI1 proteins are expressed in cisplatin-resistance cancer cell lines, suggesting their role in chemoresistance^[74]^. Notch3, which activates the NOTCH signaling pathway, is overexpressed in over 20% of high-grade serous ovarian cancer tumors, and its upregulation is correlated with tumor recurrence, chemoresistance, and poor prognostic outcome^[75,76]^. Notch3 activation has been suggested to be involved in chemoresistance by upregulating expression of stem cell markers such as nanog, OCT4, kruppel-like factor 4 (KLF4), reduced expression-1 (REX1), replication timing regulatory factor 1 (RIF1), sal-like protein 4 (SALL4), and nucleus accumbens-associated 1 (NAC1)^[75]^. Interestingly, over 30% of high-grade serous ovarian tumors are associated with PTEN loss^[77]^, which subsequently triggers the activation of the PI3-K/*protein* kinase B (AKT) pathway, leading to uncontrolled cell cycle progression, diminished apoptosis, and increased metastatic disease in ovarian cancer^[78]^. D-116883, a PI3-K inhibitor, inhibits phosphorylation of AKT that results in G0 cell cycle arrest, eventually causing apoptosis in the A2780 platinum-resistant cell line^[79]^. In addition, inhibitors of PI3-K/AKT signaling in combination with carboplatin have been shown to trigger apoptotic cell death *in vitro* and reduced ovarian cancer cell tumorigenesis *in vitro* and *in vivo*^[80]^. Levels of a PI3-K downstream target, phospho p70-ribosomal protein S6 kinase beta-1 (S6K), have been shown to be significantly higher in ascites of ovarian cancer patients who did not respond to subsequent chemotherapy^[74]^. Therefore, activation of the PI3-K pathway is potentially involved in acquired platinum resistance in ovarian cancer^[81,82]^. As we mentioned earlier, ovarian CSCs are characterized by an activated NF-κB pathway and enhanced secretion of inflammatory cytokines^[40,83]^. Aurora A kinase (Aurora A), a key cell cycle protein, which has a functional role in mitosis and meiosis, is overexpressed in ovarian cancer^[84]^. Aurora A inhibitors reduce cell proliferation in EOC CSCs by inducing cell cycle arrest and attenuating NF-κB activity^[85]^. Thus, inhibiting various signaling pathways directly or indirectly can ameliorate tumorigenesis and prevent cancer recurrence.

## Combination regimens: chemotherapy and CSC-targeted therapies

Two potential alternative therapeutic approaches could be used in ovarian cancer to eliminate CSCs. In the first approach, CSCs would be differentiated into non-CSCs followed by treatment with chemotherapy; while in the second approach CSCs are directly targeted by inhibiting the CSC-associated signaling pathways. For example, all-trans retinoic acid (ATRA), a vitamin A derivative that is used as a stem cell differentiation therapy in acute promyelocytic leukemia, contributes to the high remission rate in these patients^[86]^. ATRA is known to play an important role in cellular proliferation, differentiation, and apoptosis^[87]^. In ovarian cancer, ATRA has been shown to suppress ALDH1 expression resulting in the attenuation of CSC-like properties in ALDH^+^ cells *in vitro* and *in vivo*^[88,89]^. These results suggest that ATRA could be considered as a differentiation therapy for ovarian CSC. Several agents such as metformin and Wnt and ALDH1A inhibitors have been evaluated as potential CSC-targeted therapies. Metformin, a drug that improves glucose tolerance in type II diabetes mellitus, exhibits a favorable effect in various malignancies including ovarian cancer by attenuating tumor growth, potentially due to its suppressive effect on CSCs^[90,91]^. Low doses of metformin have been shown to selectively inhibit CD44^+^ and CD117^+^ CSCs in SKOV3 and A2780 ovarian cancer cell lines^[92]^. Furthermore, metformin treatment suppressed ALDH^+^ ovarian CSCs and was additively effective when combined with cisplatin^[62]^. In a phase II trial of 38 ovarian cancer patients with peritoneal dissemination, the median overall survival was 57.9 months after administration of the anticancer drug plus metformin before and after surgery. Tumor burden was reduced 2.4-fold, and increased sensitivity to cisplatin *ex vivo* was demonstrated in tumors established from ALDH^+^/CD133^+^ CSCs following metformin treatment^[93]^. Different Wnt pathway inhibitors have been evaluated in ovarian cancer, including salinomycin, a carboxylic acid polyether ionophore antibiotic, which reduces the activity of ABC transporters, resulting in reduced CSC chemoresistance. Treatment with a combination of paclitaxel and salinomycin exhibited growth inhibition of CD44^+^/CD117^+^ cells in three dimensional-cultured OVCAR3 cells^[94]^, suggesting this combination to be a promising regimen to be evaluated in clinical trials. Given that ALDH activity is a functional marker of ovarian CSCs, several groups have attempted to develop ALDH inhibitors. A recent report revealed that novel pan-ALDH1A family selective inhibitors preferentially target CD133^+^ ovarian CSCs. These inhibitors upregulate the expression of mitochondrial uncoupling protein (UCP) 1 and 3 and reduce oxidative phosphorylation capacity, resulting in cell programmed necrosis (necroptosis)^[95]^. Given that chemoresistant cells overexpress anti-apoptotic proteins, triggering alternative cell death pathways, such as necroptosis, can lead to better therapeutic outcomes^[96]^. The combination of cisplatin or carboplatin with an ALDH1A inhibitor caused a significant tumor shrinkage in subcutaneous, intraperitoneal, and PDX models of ovarian cancer^[95]^. Treatment with CM37, a small molecule with inhibitory activity against ALDH1A1, suppressed spheroid proliferation of ovarian cancer cells and reduced expression of OCT4 and SOX2 in ALDH^+^ cells in ascites and ovarian cancer cell lines^[97]^. Importantly, downregulation of only one isozyme from ALDH1A subfamily has been shown to upregulate others, potentially as part of compensatory mechanism, suggesting that pan-ALDH1A inhibition is required to decrease cancer stemness and self-renewal^[95]^. In addition, disulfiram, an ALDH1/2 inhibitor, exerts its anticancer effect by inhibiting ALDH enzymatic activity. This drug promotes the accumulation of reactive oxygen species (ROS) and oxidative stress, subsequently reducing expression of ovarian ALDH^+^/CD133^+^ CSC and enhancing cisplatin-induced apoptosis *in vitro* and *in vivo*^[98,99]^. Daidzin, an ALDH2 inhibitor, demonstrated no significant toxicity in ovarian cancer cells and did not affect the number of CD133^+^ cells *in vitro*. Moreover, this drug showed no therapeutic effect in combination with cisplatin *in vivo*^[95]^, suggesting that ALDH2 is not associated with cancer stemness and chemoresistance.

## Clinical studies

To summarize the novel targeted therapies that are currently being evaluated in clinical studies, a search of the clinicaltrials.gov database was conducted for active trials recruiting as of June 2020. The keywords “resistant” or “targeted therapy” in combination with “recurrent ovarian cancer” were used. A manual search to identify targeted therapies-based clinical trials was performed as well. The types of identified targeted therapies in clinical studies can be broadly classified into four types: (1) immune checkpoint inhibitors; (2) angiogenesis inhibitors; (3) PARPi; and (4) other tyrosine kinase inhibitors [Table 2]. Immune checkpoint inhibitors include program cell death protein 1 (PD-1), program cell death-ligand 1 (PD-L1), program cell death-ligand 2 (PD-L2), and cytotoxic T-lymphocyte-associated protein 4 (CTLA-4) inhibitors. PD-L1 and PD-L2 are highly expressed on CSCs of various cancers^[100]^. The use of anti-PD-L1 immune checkpoint inhibitors targets these ligands and prevents the escape of CSCs from cell death^[101]^. The category of angiogenesis inhibitors that are currently in clinical studies include bevacizumab, sevacizumab, apatinib, cediranib, pazopanib, tivozanib, and VB-111. Anti-angiogenic therapy can induce hypoxia-inducible factor (HIF-1A), which subsequently causes upregulated VEGF production and increased CSCs^[102]^. Therefore, a combination of anti-angiogenic drugs and CSC-targeted therapies may provide promising treatment options^[103]^. The FDA-approved PARPi that are currently evaluated in clinical trials include olaparib, niraparib, and rucaparib. Furthermore, clinical trials of talazoparib, a novel PARPi approved for use in breast cancer, are being conducted for recurrent ovarian cancer patients as well. Treatments with PARPi in ovarian cancer cells *in vitro* and *in vivo* resulted in enrichment of CD133^+^ and CD117^+^ CSC, suggesting a need for a combination regimen with CSC-targeted therapies^[71]^. A group of tyrosine kinase inhibitors is comprised of checkpoint kinase 1 (CHK1), ataxia-telangiectasia and Rad3 related protein (ATR), and WEE1 G2 checkpoint kinase (WEE1) inhibitors, which play a pivotal role in DNA damage repair response. These drugs have exhibited an inhibitory effect on CSCs in various cancers *in vitro* and *in vivo*, but their effect on ovarian CSCs has not been evaluated^[104]^. In addition, ongoing clinical trials evaluating the effects of different agents include PI3-K inhibitors, AXL receptor tyrosine kinase (AXL) inhibitors, microtubule stabilizers, mitogen-activated protein kinase (MEK) inhibitors, TLR8 agonists, and benzamide histone deacetylase inhibitors. PI3-K inhibitors used in cisplatin-resistant ovarian cancer cell lines exhibited reduction in CD44variant6, CD117, ALDH1A1, and Snail expression^[105]^. Interestingly, in breast cancer, inhibition of AXL increased CSC chemosensitivity^[106]^, whereas histone deacetylase inhibitors manifested preferential targeting of breast CSCs, suggesting a potential therapeutic effect in ovarian CSCs as well^[107]^. In addition, a recent study of high-grade serous ovarian cancer revealed that an MEK1/2 inhibitor, trametinib, arrests cell proliferation but also enriches cancer stemness, suggesting a need for a combination regimen with CSC-targeted therapy^[108]^.

**Table 2:** Clinical trials for platinum-resistant recurrent ovarian cancer

	Identifier	Function	Phase
Immune checkpoint inhibitor			
Durvalumab	NCT03026062	Programmed cell death ligand 1 inhibitor	2
	NCT03699449		2
	NCT02431559		1/2
	NCT02764333		2
	NCT04019288		1/2
	NCT02963831		2
	NCT02811497		2
Pembrolizumab	NCT02608684	Targets programmed cell death protein 1 receptor	2
Atezolizumab	NCT03363867	Targets programmed cell death ligand 1 (PD-L1)	2
TSR-042	NCT03574779	Anti-PD1 antibody	2
Tremelimumab	NCT02953457	Activates the immune system by targeting CTLA-4	2
	NCT03026062		2
Ipilimumab	NCT03508570	Activates the immune system by targeting CTLA-4	1
Avelumab	NCT02580058	Targets the protein programmed death-ligand 1 (PD-L1)	3
Anti-angiogenic inhibitor			
Apatinib	NCT04348032	Vascular endothelial growth factor receptor-2 (VEGFR2) inhibitor	2
Cediranib	NCT03699449	Vascular endothelial growth factor inhibitor	2
Anlotinib	NCT04376073	c-MET/TIE-2/VEGFR inhibitor	2
Tivozanib	NCT01853644	Oral VEGF receptor tyrosine kinase inhibitor	2
Bevacizumab	NCT03093155	VEGF receptor tyrosine kinase inhibitor	2
Sevacizumab	NCT03763123	VEGF receptor tyrosine kinase inhibitor	1
VB-111	NCT03398655	An anti-angiogenic gene therapy	3
Regorafenib	NCT02736305	Dual-targeted VEGFR2-TIE2 tyrosine kinase inhibition	2
Pazopanib	NCT01402271	Selective multi-targeted receptor tyrosine kinase inhibitor that blocks tumor growth and inhibits angiogenesis	1/2
PARP inhibitor			
Olaparib	NCT02889900	PARP inhibitor	2
	NCT04633239		1
	NCT03117933		2
	NCT02898207		1
	NCT03161132		2
	NCT03699449		2
	NCT03314740		2
	NCT02502266		2/3
Niraparib	NCT04376073	PARP inhibitor	2
	NCT03955471		2
	NCT04502602		1
	NCT03586661		1
	NCT04217798		2
	NCT03944902		1
	NCT01227941		1
Rucaparib	NCT03552471	PARP inhibitor	1
Talazoparib	NCT03330405	PARP inhibitor	2
Other tyrosine kinase inhibitor			
BAY1895344	NCT04267939	Ataxia-telangiectasia and Rad3 related protein (ATR) inhibitor	1
Prexasertib	NCT03414047	CHK1 inhibitor	2
Adavosertib	NCT03579316	WEE1 G2 checkpoint kinase (WEE1) inhibitor	2
Copanlisib	NCT03586661	Phosphatidylinositol-3-kinase (PI3K) inhibitor	1
TP-0903	NCT02729298	Targets the AXL (derived from the Greek word “anexelekto”, meaning uncontrolled) receptor tyrosine kinase	1
AVB-S6-500	NCT03639246	Targets the AXL (derived from the Greek word “anexelekto”, meaning uncontrolled) receptor tyrosine kinase	1/2
Ixabepilone	NCT03093155	Stabilizes microtubules	2
	NCT02595892		2
Cobimetinib	NCT03363867	MEK inhibitor	2
	NCT02101775		2
Motolimod	NCT02431559	Toll-like receptor 8 (TLR8) agonist	1/2
Entinostat	NCT03924245	Benzamide histone deacetylase inhibitor	1/2

## Conclusion

Ovarian CSCs are a population of cells that are often enriched in residual tumors following initial treatments with conventional chemotherapy. These CSCs are directly associated with acquired chemoresistance in ovarian cancer. Various studies have attempted to assess CSCs as prognostic markers using various laboratory techniques such as qPCR, immunochemistry, and FACS, to quantify mRNA and protein expression levels or enzymatic activity. This approach (using different techniques for evaluation) has introduced a lot of variations as mRNA level does not necessarily correlate with protein level or enzymatic activity. In addition, the ALDH1A antibody extensively used for immunochemical analysis does not differentiate between ALDH1A1, 1A2, and 1A3 isozymes. Thus, a more rigorous experimental design is needed to evaluate single markers and dual CSC marker combinations to insure reproducible results. A FACS-based assay for ALDH activity is affected by cell confluence and cell number used in each assay, raising the importance of standardized protocols to ensure consistent measurements amongst research groups.

Given the CSC importance in prognosis and disease progression, the molecular biology of ovarian CSCs needs to be further elucidated to design novel CSC-targeted therapies. Targeted therapies against CSCs have the potential to reduce tumor growth and improve patient prognosis. On the basis of recent findings, the combination of chemotherapy and CSC-targeted therapy may be one of the most promising anticancer treatments for ovarian cancer. Currently, many clinical trials are ongoing to evaluate conventional chemotherapy in combination with various targeted therapies in platinum-resistant ovarian tumors, which potentially will change the treatment strategy for patients with recurrent ovarian cancer. Finally, assessing how novel targeted therapies affect the various CSC populations is required to rationally design new treatment regimens.

## Declarations

### Copyright

© The Author(s) 2020.
